# PsyAPP: The Development of a Mobile Application for Effective Health Management in Mentally Ill Patients

**DOI:** 10.3390/jcm15020894

**Published:** 2026-01-22

**Authors:** Marta Llorente-Alonso, Cristina García-Ael, Gabriela Topa, Ana Fernández-Araque, Lourdes Jiménez-Navascués, Mª Ángeles Martínez-Casado, Irene Garcés Carretero, Andrea Cuervas-Mons Tejedor

**Affiliations:** 1 Faculty of Health Science, University of Valladolid, 42005 Soria, Spain; anamaria.fernandez@uva.es (A.F.-A.); lourdes.jimenez@uva.es (L.J.-N.); acuervasmons@saludcastillayleon.es (A.C.-M.T.); 2 Gerencia de Asistencia Sanitaria del Área de Salud de Soria, Complejo Hospitalario de Soria, Gerencia Regional de Salud de Castilla y León (Sacyl), Pº Santa Bárbara s/n, 42005 Soria, Spain; mamartinezc@saludcastillayleon.es (M.Á.M.-C.); igarces@saludcastillayleon.es (I.G.C.); 3 Department of Social and Organizational Psychology, Faculty of Psychology, National Distance Education University (UNED), C/Juan del Rosal, 10, 28040 Madrid, Spain; cgarciaael@psi.uned.es (C.G.-A.); gtopa@psi.uned.es (G.T.)

**Keywords:** mobile applications, digital mental health, psychiatry, health management, treatment adherence, mental health nursing care

## Abstract

**Background/Objectives**: In recent decades, new technologies have been progressively integrated into various areas of mental health care. Mobile applications are potentially effective tools that allow psychiatric patients themselves to access self-management resources and tools within the community setting. Mental health nursing plays a key role in enabling patients to take an active role in their care and in promoting activities that foster their involvement and empowerment. The primary aim of this pilot study was to develop the PsyAPP mobile application to support both nurses and individuals with mental illness in managing care and improving health outcomes, and to assess its feasibility within a real-world clinical setting. **Methods**: A mobile application (PSYAPP) and a complementary web-based nursing management platform were designed and implemented. A total of 20 psychiatric patients enrolled in a partial hospitalization program in Soria (Spain) participated. Participants were assigned to experimental (app users) and control groups. Psychological empowerment, global functioning, and suicide risk were assessed before and after the intervention. **Results**: Patients who used the application showed significantly greater psychological empowerment (*W* = 2.04, *p* ≤ 0.04) compared with the control group. Statistically significant improvements were observed in psychological, social, and occupational functioning. Regarding suicide risk, no statistically significant changes were detected between pre- and post-intervention measurements in either group. Overall, PSYAPP demonstrated feasibility and potential utility as an innovative tool to support mental health care follow-up. **Conclusions**: This study developed and implemented a mobile application designed to enhance mental health care by supporting both patients and psychiatric nurses. Results showed significant improvements in global functioning in both the app and control groups, suggesting that rehabilitative treatment contributed to overall progress. Suicide risk did not significantly change within groups, although improvements were seen in the full sample, likely due to clinical care rather than app use. Only the experimental group demonstrated increased psychological empowerment, indicating that the app may effectively enhance patient engagement and involvement in their own care.

## 1. Introduction

Mobile health approaches that support nursing care through mobile phones may help overcome some of the barriers associated with face-to-face care. Technological innovations referred to as “mHealth” or “e-health” describe the delivery of healthcare services via electronic means through the Internet, using a variety of devices, including mobile phones [1]. Specifically, the use of mobile applications and the Internet in mental health is known as e-mental health. As Hollis et al. suggested, the use of technology in mental health care has the potential to represent a shift in psychiatric services. Moreover, it may support patients in taking a more active role in managing their illness and in having greater choice regarding their treatment and care [2].

In recent decades, the use of new technologies, such as virtual reality, mobile applications, and generative artificial intelligence, has been increasingly incorporated into various areas of mental health care [3]. Evidence suggests that smartphone-based psychological interventions can effectively reduce symptoms of anxiety and depression and may serve as valuable complementary tools in the management of severe mental disorders, including early psychosis [4,5]. Importantly, mobile technologies are widely accessible: recent data indicate that over 99% of individuals aged 16–74 in Spain have used a mobile phone within the previous three months [6]. Furthermore, individuals with psychosis have demonstrated willingness to incorporate smartphone-based interventions into their treatment, reporting high levels of acceptability and adherence [7].

Mobile applications, therefore, represent a convenient and potentially effective means of delivering self-management tools and supporting continuity of care in community-based psychiatric settings [8]. These technologies may enhance treatment adherence, facilitate early detection of clinical deterioration through real-time data monitoring, and support preventive interventions aimed at reducing risky or self-injurious behaviours. Hybrid care models combining traditional mental health services with asynchronous digital tools align with current evidence and offer promising strategies to improve access, personalization, and quality of care [3].

Despite this potential, important limitations persist. Many mental health applications lack robust evidence of effectiveness, and existing studies are frequently conducted by developers or institutions with vested interests in the evaluated tools [9]. Moreover, there is a notable scarcity of experimental studies, including randomized controlled trials, comparing digital interventions with standard face-to-face care.

### Background

Mental disorders are characterized by clinically significant disturbances in cognition, emotional regulation, or behaviour and are frequently associated with functional impairment and reduced quality of life [10]. Globally, mental disorders affect approximately one in eight individuals, with anxiety, depressive disorders, and severe mental illnesses such as schizophrenia and bipolar disorder contributing substantially to disability and premature mortality [10,11].

Treatment adherence remains a critical challenge in mental health care. Evidence indicates that medication nonadherence rates in serious mental illness range from 44% to 56%, leading to poorer clinical outcomes and increased healthcare utilization [12,13]. Social support has been identified as a key protective factor, with higher perceived support associated with improved adherence and reduced psychological distress [14,15]. However, maintaining engagement and continuity of care remains difficult, particularly in community-based settings where patient monitoring is limited.

Individuals with severe mental illness often experience increased vulnerability to stress, reduced functional autonomy, difficulties in social and occupational integration, and a higher prevalence of physical comorbidities compared to the general population [16,17]. These factors contribute to markedly increased mortality rates, largely attributable to cardiovascular disease and cancer, and highlight the need for comprehensive, continuous, and coordinated care models [10].

Information and communication technologies (ICTs), including mobile health interventions, have demonstrated potential benefits in supporting self-management, organization of daily activities, and adherence in individuals with chronic and severe mental disorders [18]. Prior research has shown that mHealth interventions may improve depressive symptoms, illness-related beliefs, and engagement with care, particularly in high-risk populations [4,18,19]. Nonetheless, findings across studies remain heterogeneous, and robust evidence from large experimental trials is still limited.

Within this context, mental health nursing plays a central role in promoting recovery-oriented care, enhancing patient empowerment, and supporting adherence and self-management. Nursing practice emphasizes individualized care, patient engagement, and the development of autonomy through therapeutic relationships and ongoing monitoring. Empowerment, defined by the World Health Organization as the process by which individuals gain greater control over decisions affecting their health [20], aligns closely with contemporary nursing models and collaborative approaches to care [21].

Given the increasing complexity of mental health needs and the persistent challenges related to adherence and continuity of care, innovative nursing-led interventions supported by digital technologies may represent a valuable strategy to enhance patient engagement, empower individuals, and improve clinical outcomes. Crucially, the literature reveals a significant gap regarding mobile applications specifically managed by nursing teams, particularly those designed to support mental health nursing care, enhance patient empowerment, and promote treatment adherence.

In response to this gap, the present study introduces an innovative, nurse-managed mobile application designed to support mental health nursing care within a partial hospitalization context. Unlike most existing digital mental health tools, this application is actively integrated into nursing practice, enabling nurses to monitor patients, support self-management, and foster psychological empowerment. Through a pilot quasi-experimental design, this study aims to evaluate whether a nursing-led digital intervention can enhance patient engagement, improve adherence, and support clinical follow-up, thereby contributing novel evidence to the field of digital mental health nursing.

The primary aim of this pilot study was to develop the PsyAPP mobile application to support both nurses and individuals with mental illness in managing care and improving health outcomes, and to assess its feasibility within a real-world clinical setting.

Furthermore, the study aims to assess whether patients using the “PsyApp” application, in comparison to those receiving only face-to-face care, demonstrate better health outcomes in the following areas:

- Greater psychological empowerment;
- Reduced suicide risk;
- Higher scores in the assessment of psychological, social, and occupational dimensions of mental health.

## 2. Materials and Methods

### 2.1. APP Development

The mental health nurse specialist, based on clinical assessment, decides which users to register in the app and provides them with access credentials.

#### 2.1.1. Features of the Nursing Web Platform

From the consultation computer, the nurse enters data that each participant will later be able to view on their mobile device. The nurse accesses a private area on the platform to register each patient and initiate the app’s use.

**Figure 1:**
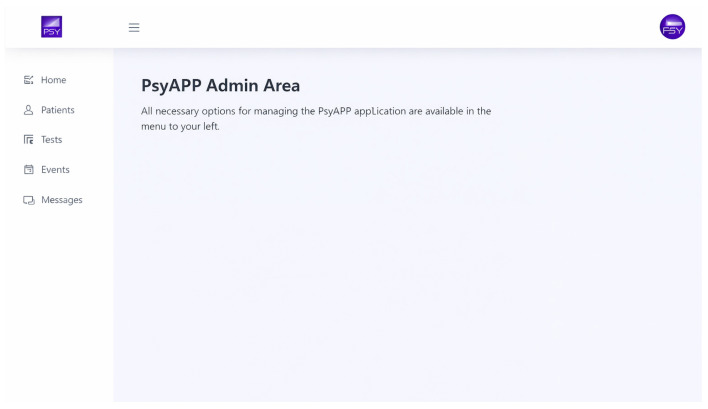
PSYAPP web domain: http://www.psyapp.es.

The platform includes the following sections:

- **Patients:** Used to generate user login credentials and input clinical data such as weight, height, BMI, blood pressure, heart rate, waist circumference, and oxygen saturation.
- **Tests:** Enables the nurse to assign questionnaires or clinical scales to patients, which they can complete online.
- **Events:** Allows the scheduling of alerts for upcoming nursing and psychiatric appointments. Medication reminders can also be set up, so the patient receives notifications on their mobile phone at the time of medication administration.
- **Messages:** Through this section, nurses can communicate with patients and check whether messages have been received from them (see Figure 1).

#### 2.1.2. Features of the Mobile Application (Available on Google Play Store)

**Figure 2:**
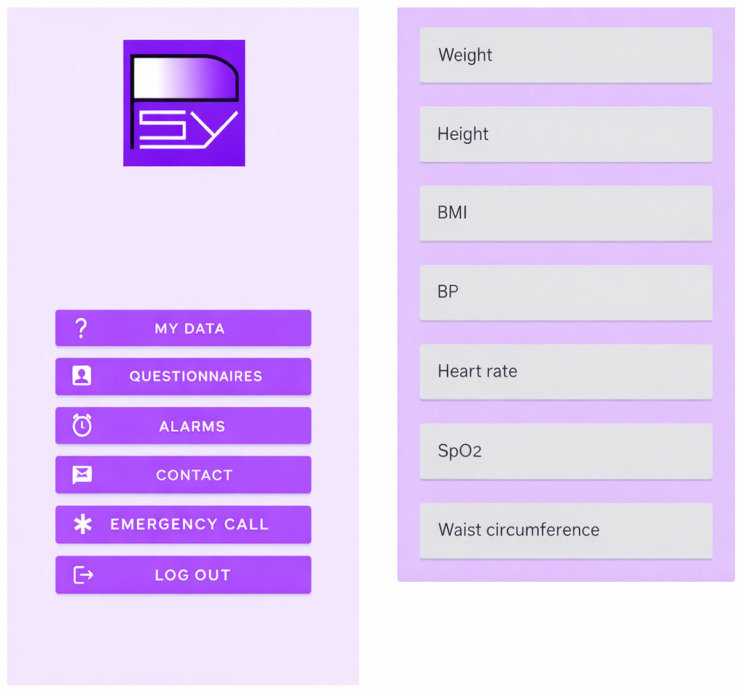
PSYAPP mobile application: Main screen and access to MY DATA. Note: BMI = body mass index; BP = blood pressure; SpO_2_ = Oxigen Saturation.

The user downloads the app and logs in to their private area:

(a) Access to Personal Data Section (See Figure 2)

The patient can view clinical and anthropometric data recorded by the nurse, including vital signs.

**Figure 3:**
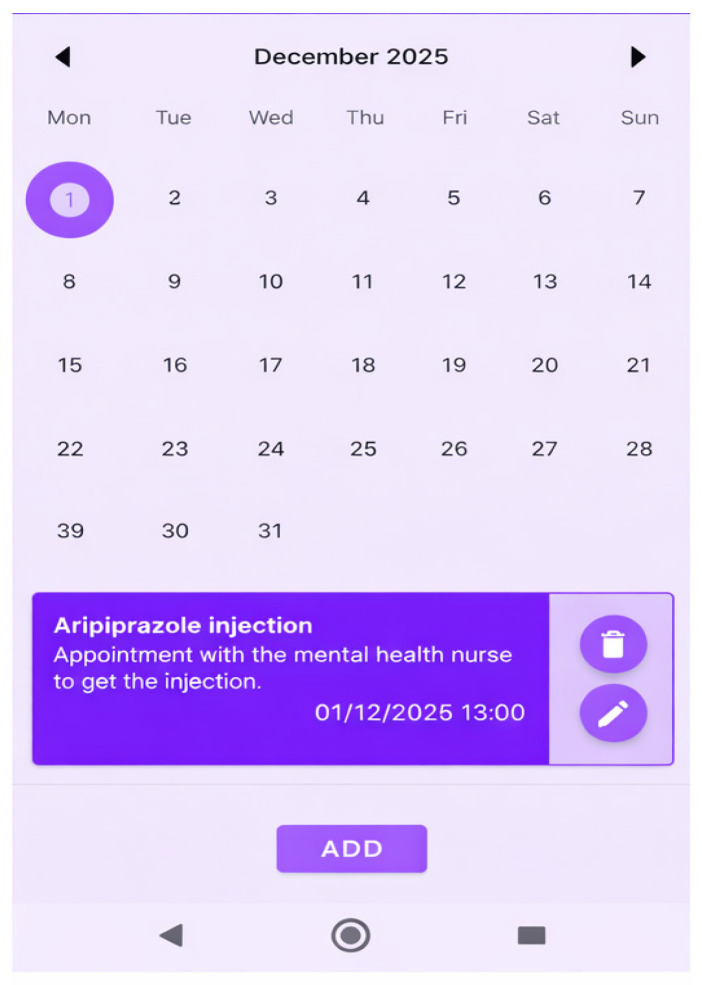
PSYAPP mobile app: Appointment and medication alerts section. Therapeutic adherence program.

(b) Appointment and Medication Reminders-Therapeutic Adherence Program (See Figure 3)

A monthly calendar is displayed where the patient can view scheduled mental health appointments. The patient receives alert notifications both the day before and on the day of their appointments with the nurse and psychiatrist. A list of currently prescribed oral medications is provided. When medication is taken, the patient can mark the task as completed using checkboxes.

**Figure 4:**
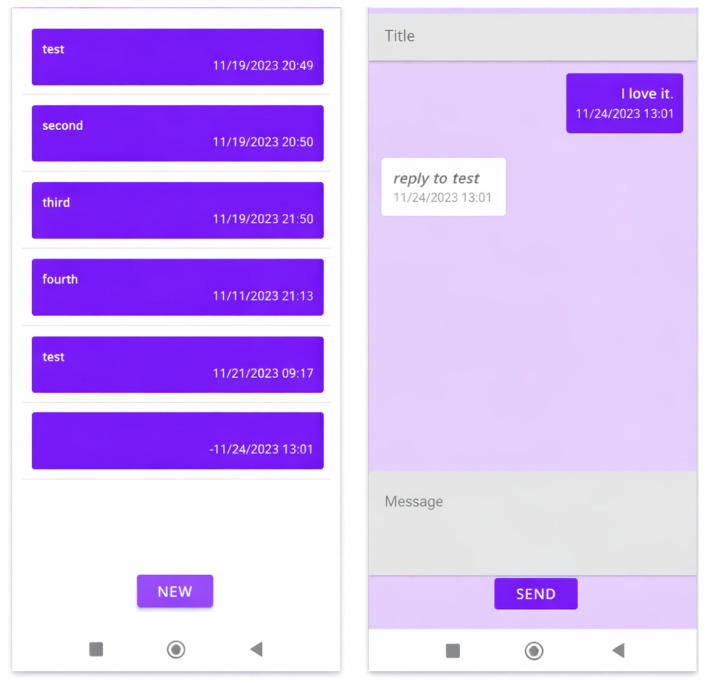
PSYAPP mobile application: Contact with the mental health nurse. Risk prevention/prodromal detection program. Note: Conversation with a patient.

(c) Contact with Mental Health Nurse-Risk Prevention and Prodromal Symptom Detection Program (See Figure 4)

Includes a non-instant messaging section for the patient to contact their nurse regarding questions or concerns. Patients are informed of the available response hours. This section is particularly useful for the early detection of prodromal symptoms.

**Figure 5:**
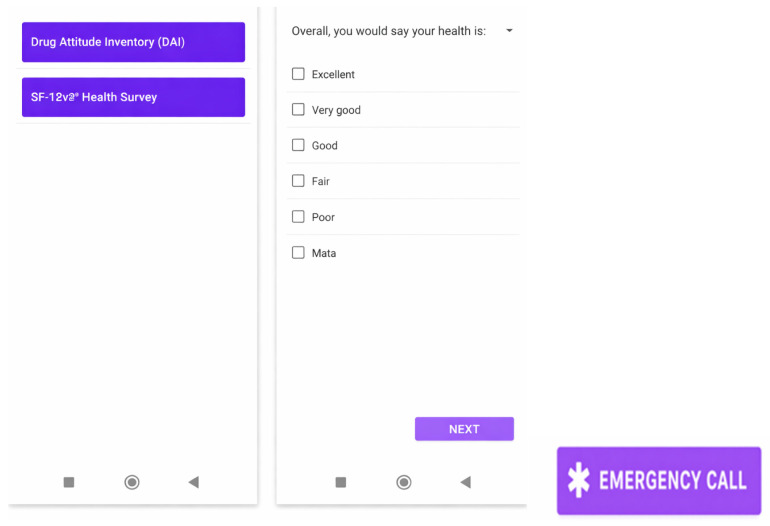
PSYAPP mobile application: Button to call 112 in case of emergency and section for periodic monitoring and control. Monitoring and periodic control program.

(d) Panic Button—Emergency Contact (112) (See Figure 5)

A dedicated button is available for direct dialling of emergency services in urgent situations.

(e) Monitoring and Periodic Assessment Section—Ongoing Monitoring Program (See Figure 5)

Includes targeted questionnaires to assess the patient’s physical, emotional, and psychiatric state every three months. In the present study, suicide risk and patient empowerment are evaluated. Empowerment is assessed through variables such as perceived control over the illness, choice of treatment setting, access to information, support from non-healthcare sources, sense of usefulness, spirituality, use of complementary therapies, and acceptance of the illness.

It is important to emphasize that the questionnaires included in the monitoring and social support programs can be modified over time via the nursing web platform. This flexibility adds scientific value to the intervention, allowing periodic evaluation of different variables and thereby facilitating improved healthcare delivery. It also enables the longitudinal assessment of key parameters related to both physical and mental health.

### 2.2. Research

#### 2.2.1. Research Design

A quasi-experimental design was employed, involving a non-equivalent control group with pre and post-intervention measures. Outcomes were assessed three months after the intervention (implementation of the app). The data collection months were from January to April 2025. The three-month follow-up period was selected because it corresponds to the predefined implementation and testing phase of the mobile application. This study was designed as a pilot study; its primary aim was not to generalize the results, but to preliminarily assess the feasibility and acceptability of the mobile application, as well as to identify potential technical or usability issues, in order to refine the application prior to larger-scale clinical studies.

Therefore, this project was conceived as a pilot evaluation of a mobile application under real-world clinical conditions. Several methodological challenges were identified that must be addressed prior to conducting studies with larger samples, such as randomized controlled trials. A quasi-experimental pre–post design was deliberately selected, as participant recruitment was based on convenience sampling within a routine clinical setting, making randomization unfeasible.

Although this design entails limitations in terms of internal validity, it allows for the assessment of feasibility, acceptability, and preliminary effects of the intervention in a naturalistic context, thereby enhancing external validity. The findings derived from this pilot study should therefore be interpreted as exploratory and hypothesis-generating, serving to inform the design and implementation of future controlled studies with larger samples and more robust methodological controls.

#### 2.2.2. Participants

The study included 20 patients receiving psychiatric treatment through the partial hospitalization program of the Psychiatry and Mental Health Service in the province of Soria. Given that the Psychiatry Partial Hospitalization Program comprises 20 patients who attend daily rehabilitative therapy sessions, this setting was considered ideal for conducting the pilot study. Daily contact with participants minimized the risk of dropout and facilitated ongoing feedback on potential issues and areas for improvement in the mobile application, supporting the evaluation of its feasibility, usability, and acceptability in a real-world clinical context.

The intervention and control groups were comparable with respect to key clinical characteristics. All participants had severe and long-standing mental disorders, which were clinically stabilized at the time of study inclusion. All participants were receiving psychopharmacological treatment as part of their usual care, including antidepressants, antipsychotics, and/or anxiolytics or hypnotics. No differences in standard clinical management or pharmacological treatment were observed between groups during the study period. Participants in the intervention group did not receive any additional or preferential clinical benefits for agreeing to participate in the study beyond access to the mobile application.

The study was conducted in accordance with ethical standards and data protection regulations. All participants provided written informed consent, and the study protocol was approved by the relevant institutional ethics committee. Data collected through the mobile application were anonymized, securely stored, and accessible only to authorized nursing professionals, in compliance with applicable data protection regulations.

#### 2.2.3. Inclusion and Exclusion Criteria

Participants were required to be over 18 years of age and to be proficient in the use of touchscreen smartphones and internet-based applications.

Eligible patients had to be diagnosed with a chronic mental disorder and be currently under treatment and follow-up by the Psychiatry and Mental Health Unit in Soria (Spain). Specifically, patients diagnosed with the following psychiatric conditions, according to the ICD-10, were considered eligible:

- Schizophrenic disorders
- Schizotypal disorders
- Persistent delusional disorders
- Induced delusional disorders
- Schizoaffective disorders
- Other non-organic psychotic disorders
- Bipolar disorder
- Severe depressive episode with psychotic symptoms
- Recurrent severe depressive disorders
- Personality and behavioural disorders in adults

Patients with mental disorders due to substance use and organic mental disorders were excluded from the study.

#### 2.2.4. Selection Procedure

Gender may contribute to heterogeneity within the sample. Considering potential bias and the digital divide in the design, implementation, and use of information and communication technologies (ICT), efforts were made to ensure a balanced distribution of male and female participants across the intervention and control groups. Gender balance was monitored during recruitment to avoid major imbalances between groups, rather than through formal randomization. Participants were assigned to the study groups following a non-equivalent control group design, without randomization, while maintaining gender balance as a matching criterion.

#### 2.2.5. Treatment Groups

- Experimental Group: Participants used the “PSYAPP” and received mental health nursing care through digital tools.
- Control Group: Participants received standard face-to-face healthcare without using ICT.

#### 2.2.6. Variables and Instruments

- Sociodemographic data: Age, gender, ICD-10 diagnosis, presence of support measures for individuals with mental disabilities.
- Clinical data: Weight, height, BMI, blood pressure, heart rate, oxygen saturation, and waist circumference.
- Empowerment, suicide risk, and GAF were selected as outcome measures. Empowerment reflects the app’s potential to enhance self-management and autonomy. Suicide risk is a critical measure in this population, allowing assessment of preventive or crisis-mitigating effects. GAF provides a standardized evaluation of overall social, occupational, and psychological functioning, capturing global changes attributable to the intervention.
  ✓ Patient Empowerment Strategies Questionnaire: This self-administered scale, developed by Bulsara and Styles, uses a 5-point Likert scale [22]. Higher scores indicate greater agreement and, consequently, a higher level of empowerment. It comprises 15 items that collectively reflect a single factor: patient empowerment. The original scale subdivides this concept into domains, including patient control over illness, choice of treatment setting, access to information, non-healthcare support, sense of usefulness, spirituality, use of complementary therapies, and illness acceptance.
  ✓ Plutchik’s Suicide Risk Scale (RS), Spanish version: This 15-item dichotomous-response scale (yes/no) assigns 1 point for each affirmative answer and 0 for negative ones. The total score ranges from 0 to 15. A cut-off point of 6 is suggested, with scores equal to or greater than 6 indicating a risk of suicide. Higher scores reflect greater risk [23,24].
  ✓ Global Assessment of Functioning (GAF) Scale: This single-item tool assesses general functioning along a hypothetical continuum of mental health to illness. Scores range from 1 to 100, with higher scores indicating better overall functioning [25].

#### 2.2.7. Procedure and Evaluation Phases

Once the application was developed, the quasi-experimental pilot study began and was implemented in the following phases:

1. Both groups underwent a baseline assessment, which included sociodemographic data, psychological variables (via the aforementioned questionnaires), and physiological measures.
2. Training sessions were held to ensure participants could effectively use the app before data collection.
3. The experimental group began using the app from day one after installation.
4. After three months of app usage, outcome measures were reassessed using the same instruments.

#### 2.2.8. Data Analysis

Data analysis was performed using SPSS version 27. Descriptive statistics were calculated for all measured variables. Group comparisons (pre and post-intervention) were conducted using non-parametric tests, with significance levels assessed to determine statistical relevance by the study’s objectives.

## 3. Results

The sample consisted of 20 patients (9 in the experimental group and 11 in the control group), including 12 women and 8 men, with a mean age of 52.7 years (*SD* = 24.74). The mean age in the experimental group was 47 years (*SD* = 35.85), while in the control group it was 57.36 years (*SD* = 8.92). Gender was evenly distributed across the treatment groups, with 6 women and 3 men in the experimental group, and 6 women and 5 men in the control group.

The most prevalent diagnoses were within the schizophrenia spectrum (30% of the sample; 6 individuals) and anxiety, dissociative, stress-related, somatoform, and other non-psychotic disorders (also 30%; 6 individuals). Personality disorders were also common, accounting for 25% of the sample (5 individuals), and 3 participants (15%) were diagnosed with bipolar disorder.

Initial clinical data showed an elevated BMI in the overall sample (*M* = 30.37; *SD* = 8.5), with an average weight of 86.49 kg (*SD* = 25.07) and an average height of 152.35 cm (*SD* = 53.56). Blood pressure values were within normal limits, with a mean systolic pressure of 123 mmHg (*SD* = 23.65) and diastolic pressure of 76.82 mmHg (*SD* = 12.27). The mean heart rate was 82 bpm (*SD* = 12.02). Oxygen saturation and waist circumference data could not be analysed due to a high number of missing values.

Missing values (e.g., oxygen saturation and waist circumference) were treated using a complete-case analysis approach, and no data imputation was performed due to the pilot nature of the study and the small sample size.

Regarding the use of the mobile application and the chat function with the mental health nurse, the most frequent users were patients diagnosed with personality disorders (4 individuals) and one patient with an anxiety disorder. The messages received via the chat function concerned questions about their condition, notifications of absence from the program, or other practical issues arising in day-to-day participation in the Partial Hospitalization Program.

Although app logins cannot be directly tracked, web data show that participants added a total of 75 medication reminders and continue to schedule appointments and update medications. The medication reminders were the most frequently used feature, with daily confirmation of intake via task checkmarks. The messaging chat was also widely used, although usage varied considerably between participants (e.g., 118 messages for one patient versus far fewer for others). Regarding questionnaires, 75% of participants completed them; the remaining 25%, all diagnosed with Schizophrenia, declined due to high levels of mistrust and suspiciousness.

In terms of the non-parametric analysis of the data collected through the app questionnaires, covering Global Assessment of Functioning (GAF), suicide risk, and psychological empowerment, several noteworthy conclusions were drawn.

Firstly, results indicated statistically significant differences in GAF scores, reflecting improvements in psychological, social, and occupational functioning. These differences were significant in both groups (*W* = 2.22, *p* ≤ 0.02 for the control group; *W* = 2.40, *p* ≤ 0.01 for the experimental group).

Secondly, no significant differences were found in suicide risk, as measured by the Plutchik Suicide Risk Scale, between the two time points (*W* = −1.8, *p* = 0.06 for the control group; *W* = −1.6, *p* = 0.1 for the experimental group).

However, greater patient engagement in their care was observed in the experimental group. This was particularly evident through the use of the medication alarm function and improvements in psychological empowerment. Statistically significant differences in empowerment scores were found only in the experimental group (*W* = 0.001, *p* = 1 for the control group; *W* = 2.04, *p* ≤ 0.04 for the experimental group). (See Table 1).

## 4. Discussion

The main objective of this pilot study was to develop and implement a mobile application designed to support both mental health nurses and patients in the management of care processes, and to explore its potential impact on preliminary health-related outcomes. As outlined in the previous sections, the application has been successfully developed and is currently being used within the Psychiatry and Mental Health Service of Soria, receiving very positive feedback from patients. This innovation aligns with findings from other authors who have reported promising results regarding the effectiveness of digital health tools in improving health outcomes [18].

A specific exploratory objective of this pilot study was to examine whether patients using the “PsyAPP” application, compared with those receiving usual face-to-face care alone, showed preliminary differences in selected clinical and psychosocial outcomes. In particular, the study explored changes in psychological empowerment, suicide risk, and the psychological, social, and occupational dimensions of mental health.

Firstly, statistically significant improvements were observed in the Global Assessment of Functioning (GAF) in both the intervention and control groups. Given that this improvement occurred across groups, it suggests that factors other than the mobile application may have contributed to patient progress. These factors may include the rehabilitative treatment provided within the partial hospitalization program, increased clinical supervision, and the structured nature of intermediate mental health care. As such, the findings should be interpreted as descriptive and hypothesis-generating, rather than as evidence of a causal effect of the intervention.

Secondly, it is noteworthy that no significant differences were found in suicide risk, as measured by the Plutchik scale, between the two time points for each group. However, significant changes were observed when analyzing the full sample. This may suggest that the reduction in suicidal ideation is primarily due to rehabilitative and psychopharmacological treatment, as well as the stabilization of the patient within an intermediate care setting. As such, the mobile application may not play a decisive role in managing suicidal ideation or in improving overall functioning.

On the other hand, increased patient involvement in their care was evident, particularly through the use of the medication reminder feature and the empowerment questionnaire. Statistically significant differences were found only in the experimental group, supporting the hypothesis that patients using the application feel more empowered and more engaged in their treatment. This finding highlights the potential value of technological tools in helping patients feel more competent and involved in managing their illness.

This nurse-managed mobile application aligns with previous digital mental health interventions that have demonstrated potential benefits in improving treatment adherence, self-management, and patient engagement among individuals with severe mental illness [26,27]. Prior studies have shown that smartphone-based interventions can support symptom monitoring, medication adherence, and psychoeducation, particularly in populations with schizophrenia and bipolar disorder [26,27]. Similarly, mHealth tools such as MONARCA and FOCUS have been reported to support illness self-management and patient engagement through daily monitoring and feedback mechanisms [28,29]. However, most existing applications are either patient-driven or clinician-supervised without being explicitly integrated into routine nursing practice. In contrast, the present application is uniquely embedded within mental health nursing care, enabling nurses to actively monitor patients, provide timely support, and foster empowerment within a therapeutic relationship. This nurse-led approach may enhance continuity of care and patient engagement, particularly in community and partial hospitalization settings, and represents an important advancement in the integration of digital tools into nurse-led mental health care.

### 4.1. Feasibility of the Mobile Application

An important aim of this pilot study was to assess the feasibility of implementing the PsyAPP mobile application in a real clinical setting. The successful development, deployment, and routine use of the application within the Psychiatry and Mental Health Service of Soria demonstrates its practical feasibility. Patients were able to use the application throughout the study period without major technical difficulties, and adherence to key functionalities, such as medication reminders and self-monitoring features, was observed.

Furthermore, the positive feedback reported by patients supports the acceptability and usability of the application in this clinical context. These findings indicate that the integration of PsyAPP into routine mental health care is feasible and well-received, providing a strong rationale for conducting future studies with larger samples, longer follow-up periods, and more robust experimental designs to evaluate effectiveness.

### 4.2. Limitations

Given that this work is a technological innovation project that required a considerable development period by an external company, the main challenges encountered during its implementation are outlined below:

- Delays in obtaining permissions from the organization responsible for data collection caused significant delays and had consequences for the subsequent research design.
- Access issues with the application, which have now been resolved.
- Problems uploading the app to iOS due to intellectual property concerns. This issue remains unresolved and is still in progress.
- Difficulties updating patient data on the web domain, which have since been resolved.
- Notifications within the web domain, which are still in the process of being addressed.
- Issues with exporting data to Excel in a format compatible with statistical software have now been resolved.
- Alarms are displayed in the same colour, potentially causing confusion among patients regarding medical/nursing appointments and prescribed medication reminders. This was highlighted as a proposed improvement by the patients themselves.

With regard to the limitations specific to the research component of the study, it is important to emphasize the small sample size and the use of non-probabilistic sampling, as all participants were selected from the partial hospitalization program. These factors may limit the generalizability of the results to the broader population.

Given the pilot nature of the study and the limited sample size, formal baseline statistical comparisons were not performed. Instead, baseline demographic and clinical characteristics were examined descriptively to identify clinically relevant imbalances. However, future studies with larger samples should include appropriate baseline comparisons and adjusted analyses.

On the other hand, at a descriptive level, an improvement in the results can be observed. However, regarding inferential statistics, the results should be interpreted with caution due to the small sample size. We acknowledge that the number of participants should be increased, and future research studies with larger samples are needed to confirm these findings.

Furthermore, it must be acknowledged that mixed innovation-research studies are often subject to numerous external variables, which can prolong and complicate data collection, as previously discussed. A key threat to external validity in this study is the Hawthorne effect [30], whereby participants, when aware of being observed or assessed, may alter their behavior in response to perceived social expectations.

These factors highlight that, although feasibility was demonstrated, results regarding clinical outcomes should be interpreted with caution, consistent with the exploratory nature of a pilot study.

## 5. Conclusions

The PSYAPP mobile application is an innovative and appropriate tool for monitoring psychiatric patients with significant practical implications. It is recommended to continue with longitudinal studies involving a larger number of patients, to establish causal relationships, as well as to conduct randomized clinical trials and multicenter studies to enhance their external validity.

Mobile digital applications can provide patients with greater access to information and services, as well as improve the clinical management of mental health nursing care. It is important to highlight the scarcity of scientific literature addressing the use of apps aimed at motivating patients, enhancing treatment adherence, or even reducing treatment dropout rates.

## Figures and Tables

**Table 1:** Wilcoxon signed-rank tests for related samples.

	*Md* Pre-Int.	*Md* Post-Int.	*W*	*p*
**GAF**				
Total sample	50	60	**3.21**	**0.001**
Experimental group	60	70	**2.40**	**0.01**
Control group	50	60	**2.26**	**0.02**
**Suicide Risk**				
Total sample	8	4	**−2.38**	**0.01**
Experimental group	9	4	−1.6	0.10
Control group	5	4	−1.8	0.06
**Patient Empowerment**				
Total sample	29	34	**1.4**	**0.1**
Experimental group	28.5	34	**2.04**	**0.04**
Control group	28	30	0.001	1

Note: N = 20; Experimental group = 9 and control group = 11. *W* = Standardized test statistic. GAF = Global Assessment of Functioning. Pre-int. = Pre-intervention.

## Data Availability

The raw data supporting the conclusions of this article will be made available by the authors on request.

## Abbreviations

The following abbreviations are used in this manuscript:

GAF	Global Assessment of Functioning
M	Mean
SD	Standard Deviation
ICT	Information and communication technologies
BMI	Body Mass Index
